# Editorial: Systemic explanations of psychological symptoms in clinical and research practice

**DOI:** 10.3389/fpsyg.2025.1603236

**Published:** 2025-06-12

**Authors:** Lisa Chiara Fellin, Hanne De Jaegher, Michael Finn, Ellen Reijmers, Laura Galbusera

**Affiliations:** ^1^Department of Human and Social Sciences, University of Bergamo, Bergamo, Italy; ^2^Department of Logic and Philosophy of Science, University of the Basque Country, Bilbao, Spain; ^3^College of Human Medicine, Michigan State University, East Lansing, MI, United States; ^4^Interactie-Academie, Institute for Psychotherapy and Systemic Practices, Antwerp, Belgium; ^5^Klinik für Psychiatrie, Psychosomatik und Psychotherapie, Medizinische Universität Brandenburg Theodor Fontane, Rüdersdorf, Germany

**Keywords:** systemic thinking, depathologization, mental health, critical psychopathology, complexity theories, socio ecological systems, socio-relational approaches

Socio-constructionist systemic models envisage all behaviors, including symptoms, as complex and meaningful relational processes, shaped by multiple intertwined factors that are not confined within the individual and that cannot be reduced to single dimensions, nor be de-contextualized. They hence share an interpersonal and non-pathologising perspective on problems and their possible solutions, drawing on the fundamental interconnectedness of the human condition: people's lives are inextricably intertwined, and their behavior is, to a great extent, a function of the way they interact with one another.

Over the last few decades, systemic thinking has gained increasing recognition in several scientific fields for its non-reductionist, complexity-based perspectives. This Research Topic presents a diverse and international array of contributions that collectively underscore the ongoing relevance and potential of systemic approaches in understanding and deconstructing psychological symptoms. The featured articles challenge the labeling and individualistic tendencies that have long dominated mainstream psychiatry and clinical psychology, advocating instead for models that recognize the complexity, and interconnectedness inherent in mental health “disorders” offering innovative perspectives for both conceptualizing and addressing them.

## Challenging traditional explanatory models

Constant et al. overcome the disciplinary boundaries within evolutionary, cultural, and computational psychiatry, arguing that these siloed approaches limit our understanding of mental disorders. They put forward the *Evolutionary, Cultural, and Computational (ECC) model*, which seeks to integrate the three approaches by adopting a multilevel systemic perspective. To illustrate how the ECC model could provide a more comprehensive understanding of mental disorders -by accounting for both the biological and cultural dimensions and modeling their interaction computationally- they apply it to Major Depressive Disorder (MDD).

Gómez-Carrillo and Kirmayer too emphasize the importance of systemic and contextual factors in shaping mental health. They complement Constant et al.'s perspective by critiquing the reductionist tendencies within contemporary psychiatry, which often focus narrowly on neurobiological mechanisms. They explore the limitations of models constructing mental health and cognitive processes through isolated, linear causal chains. Embracing systemic thinking, they advocate for an *ecosocial systems view*, and emphasize that psychological phenomena should be understood as dynamic systems, intricately shaped by the ongoing interactions and feedback loops between individuals and their socio-cultural contexts.

Gallagher adopts an enactive approach to address the integration problem in psychiatry. Arguing against the conventional use of hierarchical levels to explain the diverse processes involved in psychiatric disorders, Gallagher propose a model based on dynamical causality and a non-hierarchicalconcept of gestalt, where processes are understood as dynamically integrated rather than operating at different levels. By applying this model to Autism Spectrum Disorder (ASD) he provides a compelling case study, demonstrating how a level free, dynamical approach can offer a holistic and accurate understanding of disorders.

Similarly, García and Arandia gain insights from enactive cognitive science integrating concepts from Gilbert Simondon's philosophy of individuation to propose a relational model of causality in psychiatry. They emphasize that mental disorders arise from the complex interplay of tensions across multiple domains—i.e., organic, sensorimotor, and social: disruptions in the sensemaking process, where individuals generate meaning through their interactions with the environment, are central to understand mental disorders. They also emphasize *transindividuality*, highlighting the role of social relations. Finally, they too advocate for therapeutic interventions that address these dynamic, interconnected processes rather than targeting isolated causes.

Rucińska and Fondelli introduce an enactive framework informed by a systemic approach for understanding how therapeutic change can be facilitated through metaphorical thinking. They conceptualize metaphors as embodied, enacted, or ecological processes, extending them beyond traditional linguistic interpretations: metaphors gain their therapeutic power through dynamic engagement and action within dialogue, rather than through static comparison or intellectual insight. By drawing on enactive cognitive science, which sees language as an embodied, interactive process, they thus offer a non-reductionist explanatory account: the therapeutic power of metaphors lies in their ability to be enacted and co-constructed in interaction, leading to transformative experiences for the client.

## The persistence of reductionism and the need for systemic re-thinking in psychiatry and mental health

While systemic approaches are gaining traction, the study conducted by Fellin et al. highlights the persistent dominance of bio-reductionist views in mainstream psychiatry. The Authors argue that the prevailing but debunked biomedical model, with its exclusive focus on nosographic classification and unsubstantiated or discarded pathophysiological hypotheses, remains overly reductionist: it neglects the complex, relational, and systemic factors at the core of psychopathology. They exemplify it by analyzing how mood disorders are constructed in mainstream psychopathology textbooks: aetiological explanations are still predominantly monadic and intrapersonal (biological), with minimal attention given to systemic and interpersonal aspects. This shows that mainstream psychopathology remains largely “resistant” to systemic thinking, continuing to prioritize the biomedical model, despite its limitations have been largely exposed.

Thoma et al. also challenge these individualistic tendencies by comparing systemic contributions with those of the German phenomenological psychiatrist Wolfgang Blankenburg, particularly his concept of the “loss of common sense” in schizophrenia. Blankenburg's approach is noted for its integration of social and familial contexts, moving beyond the individualistic focus of earlier phenomenological psychiatry: his seminal research on families of young people with schizophrenia had already identified specific structures hindering the individual's social integration and emancipation. Much of Blankenburg's work was a precursor to contemporary systemic approaches, which view mental disorders as arising from the interplay between individual, socio-relational, and cultural factors. By contrasting these two approaches, the Authors also emphasize their differences and the importance of teleological explanations, focusing on the reasons and motivations behind symptoms, rather than purely etiological causes, in accordance with previous systemic research (Ugazio et al., 2020, Fellin et al.).

## The role of social networks in explanatory and therapeutic models

The importance of social networks in the treatment and explanation of mental disorders is another central theme, particularly highlighted in the inspiring studies by Braus et al. and Hunger-Schoppe et al.. Both emphasize the complex dynamics within social networks and their impact on mental health, focusing specifically on Alcohol Use Disorders (AUD) and Social Anxiety Disorder (SAD).

Braus et al.'s cross-sectional study explores the relational factors contributing to the etiology, maintenance, and recovery from AUD. They introduce the concepts of Support Social Networks (SSN) and Craving Social Networks (CSN) to distinguish between the types of social interactions that either support recovery or exacerbate craving. Their findings reveal that individuals in full remission from AUD tend to have smaller, less negative craving networks compared to those who are not, while maintaining robust support networks. Their innovative work also highlights the ambivalence within these networks, where craving-associated relationships can provide some degree of social support, complicating the recovery process. This underscores the dual roles social relationships play in both sustaining and recovering from addiction. Hunger-Schoppe et al. emphasize the critical role of social networks in the treatment of mental disorders, particularly SAD, as they are deeply embedded in broader social networks, not isolated within individuals. The Authors introduce an Integrative Systemic and Family Therapy (ISFT) approach, which emphasizes involving various social system members—including family, friends, and colleagues—in the therapeutic process. This systemic framework recognizes that mental disorders are deeply embedded in broader social networks, not isolated within individuals. Their randomized controlled trial (RCT) demonstrates that ISFT led to significant improvements in both social anxiety symptoms and overall social functioning compared to Cognitive Behavioral Therapy (CBT). These important results highlight how mental disorders can heal within a more holistic and hence effective approach to therapy.

Both articles collectively argue for the need to incorporate a nuanced understanding of social networks into explanatory models and treatment approaches, particularly in systemic therapy, to enhance the effectiveness of interventions.

## Conclusion

All these innovative international contributions emphasize how different disciplinary traditions are now embracing a more systemic and contextual approach viewing mental distress as an understandable reaction to wider relational and societal problems, rather than situated solely “within the sufferer and their brain”. They challenge traditional individualistic and reductionist models that can often lead to misunderstanding and mistreating psychopathology and they advocate instead for more relational and contextual approaches. Different research demonstrate how mental health can be better understood and addressed when viewed through systemic lenses: they highlight the critical role of social networks, cultural contexts, and systemic integration in both the explanation and treatment of psychological symptoms and disorders. They also underscore the importance of continuing the interdisciplinary dialogue and cross-contamination that has historically enriched systemic therapy, demonstrating how integrating insights and empirical evidence across disciplines can significantly enhance our understanding and treatment of “mental disorders”. By drawing also from fields such as cognitive sciences, developmental psychology, and transcultural psychiatry, adopting a systemic framework can not only enhance our clinical practice, but also foster more empowering narratives for patients, helping to alleviate feelings of blame, guilt, and shame often associated with reductive explanations. This Research Topic thus serves as a compelling example of how interdisciplinary efforts can drive progress in these fields.

As systemic thinking continues to evolve, it holds the promise of further enhancing both research and clinical practice. This Research Topic emphasizes the enduring value of systemic approaches in psychiatry and psychology and encourages ongoing exploration and integration of these perspectives to foster more comprehensive and effective mental health care. This is in accordance with the recent UN (Pūras, 2017) and World Health Organization (2021) recognitions of a need for radical transformation of the mental health paradigm and service landscape, to better recognize and respond to the holistic needs of people who use -but are often chronicized by those very- services. Embracing a systemic perspective may pave the way for a unique opportunity toredevelop mental health services for children and adults in a way that can heal them, rather than further pathologise and disempower them.
